# Development of an Age-Appropriate Mini Orally Disintegrating Carvedilol Tablet with Paediatric Biopharmaceutical Considerations

**DOI:** 10.3390/pharmaceutics13060831

**Published:** 2021-06-03

**Authors:** Dilawar Khan, Daniel Kirby, Simon Bryson, Maryam Shah, Afzal Rahman Mohammed

**Affiliations:** 1 Aston Pharmacy School, College of Health and Life Sciences, Aston University, Birmingham B4 7ET, UK; 139140185@aston.ac.uk (D.K.); kirbydj1@aston.ac.uk (D.K.); 2 Proveca Ltd., WeWork, No.1 Spinningfields, Quay Street, Manchester M3 3JE, UK; simon@proveca.com (S.B.); maryamshah@proveca.com (M.S.)

**Keywords:** age-appropriate, paediatrics, ODMT, heart failure, biorelevant, DSC, disintegration, dissolution, flowability, PUMA

## Abstract

Owing to considerable differences observed in anatomy and physiology between paediatric subsets, it has been well established that children respond to drugs differently compared to adults. Furthermore, from a formulation perspective, there is a distinct challenge to develop a dosage form that is capable of safely, accurately, and reliably delivering the dose across the whole paediatric population. Orally disintegrating mini-tablets (ODMT) have widely been considered as an age-appropriate formulation option that possess the ability for adequate dose flexibility, avoids swallowing difficulties, and exhibits superior stability due to its solid state. Within this study, two strengths (0.5 mg and 2 mg) of carvedilol ODMT formulations were developed using an excipient composition and load that is appropriate for paediatric use. The formulations demonstrated adequate mechanical strength (>20 N) and fast disintegration times (<30 s). Dissolution profiles observed were robust and comparable to the marketed conventional tablet formulation across various parts of the gastrointestinal (GI) tract in both the fed and fasted state, signifying appropriate efficacy, quality, and performance. As such, the formulations developed in this study show potential to address the need of an ‘age-appropriate’ formulation of carvedilol, as highlighted by the European Medicines Agency (EMA) Inventory of the Needs for Paediatric Medicine.

## 1. Introduction

In Europe, a product licence (‘marketing authorisation’) is required to market a medicinal product that specifies the agreed terms of use, including medical indication, target age, dose, and route of administration [1]. However, many medicines intended for and used within the paediatric population do not have a product licence and, therefore, are often subjected to ‘off-label’ and unlicensed use. Indeed, owing to insufficient age-appropriate formulations that enable paediatric dosing and administration, many medicines are manipulated prior to use (e.g., crushing of tablets, emptying content of capsules and sprinkling over food, or changing the dosage form type), with an aim to improve patient compliance and adherence [2]. Although this serves as a vital option to deliver medicines to children when needed, many safety-related incidents have been reported, due to contamination, and purity and potency inconsistencies [3,4]. On account of such limitations and to limit the potential of unknown adverse effects of unlicensed and ‘off-label’ use, the primary expectation and preference with paediatric pharmacotherapy is to develop an age-appropriate and ready-to-administer dosage form that is licensed and commercially available [5]. This provides regulatory safeguards and ensures the formulation intended has thoroughly been evaluated in all aspects of safety and efficacy and is appropriate for paediatric use.

The benefits and limitations of current paediatric formulation platforms have been evaluated, where innovative and flexible solid oral dosage forms have shown superior acceptability among paediatric subsets [6]. Other than exhibiting greater chemical and microbial stability, flexible solid oral dosage forms including pellets, multiparticulates, sprinkles, and mini-tablets also display desirable characteristics associated with liquid dosage forms, such as enhanced dose flexibility and ease of swallowing. The World Health Organisation (WHO) has appraised flexible solid oral dosage forms including orodispersible, chewable, and soluble tablets as the most reasonable and appropriate paediatric specific dosage form [7]. An orally disintegrating mini-tablet (ODMT) combines features of both innovative and flexible solid dosage forms and exhibits all characteristics of an age-appropriate formulation, including enhanced dose flexibility, delivers an accurate dose, appropriate dosing volumes, stability, and palatability [8].

In 2007, the paediatric regulation came into force and aimed to improve the health of children by facilitating the development and availability of medicines for children [9]. The paediatric use marketing authorisation (PUMA) was introduced, with an aim to stimulate research in existing compounds that are off-patent and/or to help transform known off-label and unlicensed use into authorised use. Nonetheless, the success rate of the PUMA is below anticipated levels, with only six PUMAs being granted till date [10].

Carvedilol is a weakly basic biopharmaceutical class II model drug with a pKa value of 7.8 and aqueous solubility of 4.44 mg/L [11]. It is a third generation non-selective beta blocker that also possesses alpha blocking properties. Only a small number of randomised clinical trials have taken place in paediatrics patients with heart failure, which have shown a positive impact of carvedilol on the left ventricular function, clinical condition, and symptoms of heart failure [12,13,14,15,16,17]. Although carvedilol has been approved since 1995 and its clinical need has been established, no attempt has been made to reformulate carvedilol into an age-appropriate formulation; the only licensed medicinal form that is available is a tablet. Owing to the lack of appropriate paediatric specific formulations, specials are therefore often prescribed. The composition and excipient load, suitability, and safety of such dosage forms have not been thoroughly evaluated and therefore increase the risks of potential adverse effects. Government agencies such as the EMA have identified commonly prescribed medicines in need of age-appropriate formulations. Carvedilol for treatment of hypertension and heart failure is one of the many medicines listed within the EMA Inventory of paediatric therapeutic needs [18]. In order to satisfy the unmet need of the availability of age-appropriate and paediatric specific formulations, a modern systematic formulation development approach was perused. An excipient composition and load appropriate for paediatrics was identified, followed by compatibility studies, optimisation of blending process, and paediatric biorelevant evaluation. The selection of strengths was based off of individual child weights, followed by a ‘pick and mix’ method ensuring applicability across the whole paediatric population whilst keeping the total number of tablets administered as low as possible.

The aim of the present work was to develop an age-appropriate formulation of carvedilol, with paediatric biopharmaceutical considerations for evaluation. Formulation selection was based on API properties, target dose banding, and the suitability of a delivery system for paediatric population needs. A systematic approach including excipient screening, formulation development, characterisation, dissolution studies, and stability evaluation were conducted to generate a holistic preclinical data set. The application of the ODMT technology with paediatric biopharmaceutical consideration for evaluation aims to safely and accurately facilitate the development of more age-appropriate formulations for this vulnerable patient group and serves as a potential candidate for PUMA application.

## 2. Materials and Methods

### 2.1. Materials

Carvedilol was obtained by Acros Organics (Morris Plains, NJ, USA). Pharmaceutical grade D-Mannitol (≥98% purity) and magnesium stearate were obtained from Sigma-Aldrich (Dorset, UK), while MCC as Pharmacel 102 was obtained from DFE Pharma (Germany). Lastly, colloidal silicon dioxide (Aerosil 200) was obtained from Evonik Industries (Essan, Germany). Pepsin from porcine gastric mucosa (≥400 units/mg protein), sodium hydroxide pellets, sodium chloride, sodium acetate, and sodium monooleate were purchased from Sigma-Aldrich (Dorset, UK). Sodium taurocholate hydrate, 96%, acetic acid, maleic acid and Lecithin, 60%, eggs were obtained from Alfa Aesar (Lancashire, UK). Glyceryl monooleate as Monomuls 90-O 18 PH was kindly gifted by BASF (Ludwigshafen, Germany). Carvedilol tablets, 3.125 mg, were obtained from Bristol laboratories (Hertfordshire, UK).

For sample analysis, Acetonitrile and Methanol (HPLC-grade) were obtained from Fisher Scientific (London, UK), whereas Trifluoroacetic acid (TFA), for HPLC (≥99.0%) was purchased from Sigma-Aldrich (Dorset, UK).

### 2.2. HPLC Analytical Method Development

All samples were analysed on an Agilent 1260 series (Agilent Technologies, Santa Clara, CA, USA), equipped with a reverse-phased Eclipse plus C18, 4.6 × 150 mm, 3.5 μm column (Agilent Technologies, Santa Clara, CA, USA) was used. Separation of carvedilol was achieved using an isocratic mobile phase compromising TFA: ACN (55:45 *v*/*v*). TFA was used at a concentration of 0.1% (*v*/*v*). Flow rate was set at 1 mL/min and a wavelength of 240 nm was used for detection. A 7-point calibration curve in the range 0.19 to 12.5 mg/mL was prepared via serial dilution in methanol (1 in 2 dilution). All samples prepared for analysis were diluted so as to fall within the calibration range. Method validation was carried out following The International Council for Harmonisation of Technical Requirements for Pharmaceuticals for Human Use (ICH) Guidelines (Q2(R1)) [11].

### 2.3. Compatibility Using Differential Scanning Calorimetry (DSC)

Excipient and drug compatibility were studied using a TA DSCQ200 apparatus (TA Instruments, New Castle, DE, USA) with TA Instruments Universal Analysis 2000 software. Data was collected under nitrogen atmosphere (flow rate: 50 mL min^−1^) using sealed flat-bottomed TZero aluminium pans and pierced lids (sample mass 1–2 mg) at a heating rate of 10 °C min^−1^ in the range of 50 to 400 °C. For samples containing both the drug and excipient, 1 mg of each was measured in a weighing boat and gently mixed with a spatula for one minute before being transferred into the sample pan. All studies were conducted in triplicate and data are presented as mean ± standard deviation.

### 2.4. Particle Size Analysis

Particle size analysis was carried out via laser diffraction using a Sympatec Helos detector equipped with a Rodos dry disperser and vibrating feeder (Clausthal-Zellerfeld, Germany). A total of 1 g of sample was placed on the VIBRI/I feeder and fed through the RODOS disperser. Pressure was set at 2 bars and measuring range was set between 0–175 μm. Measurements were taken in triplicates and presented as ±SD. Volume mean diameter (VMD), X10, X50, and X90 were the values obtained. Both starting materials and final blends were analysed to give an indication of flow based on particle size and to ensure acceptable flow properties are prevalent for successful direct compression.

### 2.5. Powder Flow Measurements

Angle of repose was determined using the method outline in the U.S. Pharmacopeia monograph, <1174> [19]. A known amount of powder was poured through a 12-mm funnel maintained at a height of 2–4 cm from the top of the powder heap. Height and diameter of the powder cone was measured and the angle of repose was determined using the following equation:

tan (ϴ) = Height/0.5 Base(1)

Measurements were carried out in triplicates and presented as mean ± standard deviation.

Bulk (ρbulk) and tapped densities (ρtapped) were also used to assess powder flow. A Sotax tap density tester USP II (Allschwil, Switzerland) was used as outlined in the USP monograph, <616> [20]. A 25-mL measuring cylinder was tightly fastened onto the tester using a foam ring. A known amount of sample was then poured and the initial volume was taken at 0 taps. Further measurements were taken after every 10, 500, and 1250 taps. Measurements were carried out in triplicates and presented as mean ± standard deviation.

Carrs’s index and hausner ratios were calculated using the following equations:

Carr’s Index = (ρtapped-ρbulk)/ρtapped × 100(2)

Hausner ratio = ρtapped/ρbulk(3)

ρbulk = Mass/V0 (unsettled apparent volume)(4)

ρtapped = Mass/Vf (Final tapped volume)(5)

### 2.6. Optimising the Process of Blending for Carvedilol to Improve Content Uniformity

An Erweka AR403s Multi-Lab (Heusenstamm, Germany), equipped with a cube mixer attachment was used to mix a total of 50 g of powder that included mannitol, MCC, magnesium stearate, colloidal silicon dioxide, and carvedilol. Different speed rates, mixing times, and blending order were evaluated to analyse any consequent effects on content uniformity. In each case, magnesium stearate was added at the end and allowed to mix for a total of 1 min, whilst colloidal silicon dioxide (CSD) was added second to last and allowed to mix for a total of two minutes. A Sartorius LA220S balance (Sartorius, Göttingen, Germany) was used to accurately weigh out samples.

Content uniformity was assessed using a random sampling technique, where 10 samples of 50 ± 1 mg were taken out from different parts of each powder blend. Each sample was dissolved with 50 mL of methanol in a volumetric flask and sonicated for 30 min. Post sonication, the samples were filtered through a 0.45-μm syringe filter and analysed via High Performance Liquid Chromatography (HPLC).

### 2.7. Orally Disintegrating Mini Tablet (ODMT) Production

Optimised powder blends were identified and selected, possessing acceptable tablet properties with improved content uniformity. Two strengths of carvedilol ODMTs with a target drug load of 1 and 4% *w*/*w* and a tablet mass of 50 mg were produced using a Specac semi-automatic hydraulic press (Slough, UK) equipped with a 4-mm multi-tip (three) with concave faced punches at a compression force of 10 KN with quick release. After step wise fine-tuning of the formulation composition and tabletting parameters, two formulations for ODMT production were finalised (Table 1). The resulting ODMTs demonstrated adequate strength (>20 N) with disintegration times of less than 30 s. The need of disintegrants and flavour enhancer was eliminated due to the careful selection of excipients that provided multiple interests including palatability, compressibility, and disintegrating properties.

### 2.8. ODMT Physical Evaluation

#### 2.8.1. Hardness

A Copley TBF 100 Hardness tester (Nottingham, UK) was used to measure the force required to break the tablets produced. Hardness values were measured in Newton’s. Measurements and carried out in triplicates and presented as mean ± standard deviation.

#### 2.8.2. Friability

Friability testing was carried out using a Sotax F2 Friabilitor (AllSchwill, Switzerland) that measures the capacity of tablets to resist mechanical stress. Six tablets were carefully de-dusted using a soft brush and an initial weight was taken. Tablets were then placed in the rotating drum and rotated for 4 min at a speed of 25 rpm (total 100 revolutions). The tablets were removed, dusted, and a final weight was taken. The percentage friability was calculated using the following equation:

% Friability = (initial weight − final weight)/initial weight × 100.

#### 2.8.3. Disintegration

Disintegration testing was carried out as stated in the official USP disintegration monograph <701>. An Erweka ZT3 (Heusenstamm, Germany) disintegration tester was used. A tablet was placed in one of the six vessels and allowed to oscillate at a rate of 30 cycles per minute. In this instance, a disk was not used in order to allow for a more direct comparison of the oral cavity environment. A total of 800 mL of distilled water was used as the disintegration medium that had a constant temperature of 37 °C. Measurements were achieved for a single tablet at a time to enhance accuracy. The disintegration time was noted until there was no more residue of tablet aggregates left on the upper side of the vessel mesh. Measurements were carried out in triplicates and presented as mean ± standard deviation.

### 2.9. Adult and Paediatric Biorelevant Media Design and Development

Adult simulated gastric and intestinal media (FaSSGF, FeSSGF, FaSSIF, and FeSSIF), were prepared as presented by Jantratid et al. [21] and employed as a reference to adjust age-specific media composition. Maharaj et al. [22] defined the composition of neonatal and infant biorelevant media after investigating and evaluating age-specific changes in intra gastric parameters including bile acid, pepsin and sodium chloride concentrations, pH, and buffering capacity. A detailed summary of biorelevant media design is given in Table 2.

### 2.10. ODMT Evaluation–Biorelevant Solubility and Dissolution Studies

The saturation solubility of carvedilol in simulated dissolution media was determined according to the reported method [11]. Briefly, an excess amount of carvedilol powder was added to glass vials containing 30 mL of the respective dissolution media. The vials were then screwed and placed on a hot-plate stirrer (Stuart US152, Northants, UK) maintained at 37 ± 1 °C and continuously stirred using a magnetic stirrer at a speed of 50 rpm. Samples were withdrawn after 24 h, filtered through a 0.45-μm syringe filter, and analysed. The concentration of carvedilol in each sample was determined using HPLC. Solubility in each respective medium was carried out in triplicates and presented as mean ± standard deviation.

Sink value (S/C) was calculated as a ratio of solubility (S) to drug concentration (C) to determine whether sink conditions were achieved. A sink value greater than 3 indicates sink conditions [23]. Percentage carvedilol ionisation was calculated using the following equation (Equation (6)):

(6) $$\%\mathrm{Ionisation}=\frac{1}{1+10^{\left(\mathrm{pH}-\mathrm{pKa}\right)}}\times 100$$

where pH denotes the pH value of the dissolution medium, whilst pKa is the negative log of the acid dissociation constant of carvedilol.

Minimum dissolution volume was calculated using sink condition (Sc), determined by the ratio of solubility (S) to drug concentration (C), denoted by S/C. A value of greater than 3 is considered as sink condition [23]. All dissolution tests were carried out using an Erweka DT 126 with USP 2 paddle apparatus (Langen, Germany). Samples of 5 mL were drawn at appropriate time points (2, 5, 10, 15, 30, 45, 60, 75, 90, 120, and 180 min) and replaced by 5 mL of fresh media to maintain sink conditions. A total of 500 mL of media per vessel, maintained at 37 °C was employed with a continuous paddle speed of 50 rpm. Drug release was determined via HPLC and adjusted for cumulative drug release (%). A total of 6 replicates were taken and data presented as mean ± standard deviation.

### 2.11. Statistical Analysis

GraphPad Prism version 8 (GraphPad Software, San Diego, CA, USA) was used to carry out one-way analysis of variance (ANOVA) with a posthoc Tukey’s test to identify statistically significant differences in solubility between adult and paediatric biorelevant media. An alpha value of 0.05 was used.

## 3. Results and Discussion

Although carvedilol has been approved since 1995 and its clinical need has been established, no attempt has been made to reformulate carvedilol into an age-appropriate formulation; the only licensed medicinal form that is available is a conventional tablet, while forms available from special-order manufacturers include oral suspension. In addition, the EMA Inventory of the Needs for Paediatric Medicine has also listed carvedilol in need of an ‘age-appropriate’ formulation. To address this formulation gap, the objective of the current work was to develop two strengths (0.5 mg and 2 mg) of an age-appropriate carvedilol formulation that possess increased dose flexibility, palatability, safety, quality, and efficacy. Carvedilol is a low dose potent compound with low aqueous solubility. It is gastric stable and does not exhibit any taste concerns. For such reasons and with an aim to minimise the total number of excipients within the formulation, the selection of an ODMT was most suitable. The dose of carvedilol is based on child weight and is usually titrated when initiated. The availability of multiple strengths ensures to cover a wide range of therapeutic doses and therefore, the novel ODMT formulation is aimed at birth up to the age of 12 years.

### 3.1. Compatibility Using Differential Scanning Calorimetry (DSC)

In order to limit the total number of excipients within the formulation, one of the objectives of this study was to exploit excipient characteristics that provided multiple functions including palatability, compressibility, and disintegrating properties.

Drug and excipient compatibility is an important part of pre-formulation study, where concomitant use of excipient and active pharmaceutical ingredients (API) are assessed. Commonly used excipients in ODT formulation were scanned alongside carvedilol to determine compatibility. However, for the purpose of this study, carvedilol:excipeint compatibility is displayed for excipients that were included in the final formulation only.

**Figure 1:**
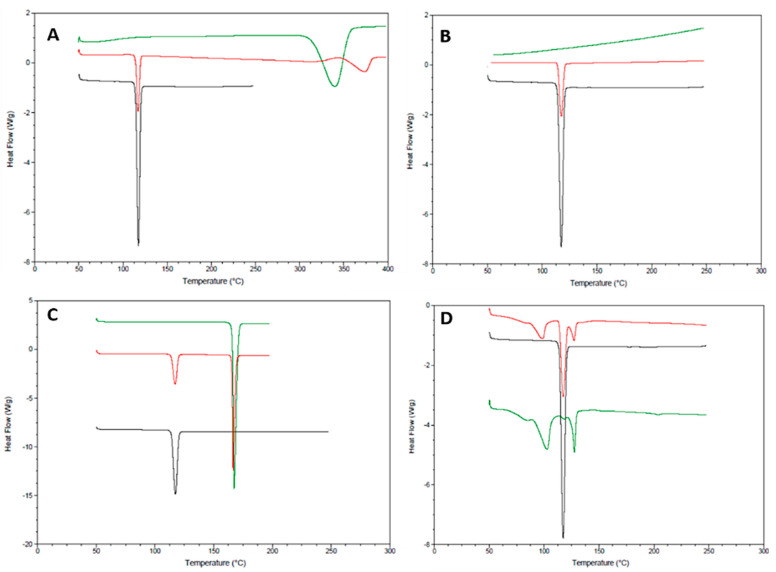
DSC thermograms showing compatibility between carvedilol and selected excipients. (**A**) MCC, (**B**) CSD, (**C**) mannitol, and (**D**) magnesium stearate. In each thermogram, the black, red, and green curves represent carvedilol, the excipient, and a 1:1 of carvedilol and excipient, respectively.

Carvedilol displayed a sharp endothermic peak at 117.33 °C, indicating its melting point and crystalline structure (Figure 1). The phase transition enthalpy value was 149.3 J/g. Polymorphism is a well-known phenomenon for fatty acids and their salts [24]. The broad melting range of magnesium stearate is typical of commercial samples because commercial magnesium stearate consists of either crystalline hydrates (di or trihydrate, or a mixture of), or a poorly crystallised anhydrate [25,26]. Magnesium stearate displayed two endothermic signals: A small one with a peak temperature of 87.92 °C (enthalpy: 19.70 J/g), and a larger one with a peak temperature of 115.03 °C (enthalpy: 274.7 J/g), where the former corresponds to dehydration and the later indicates the fusion of constituent fatty acids [27]. The DSC curve of MCC shows a broad endothermic signal between 50–125 °C (peak value: 78.51 °C and enthalpy: 37.37 J/g) corresponding to the loss of humidity and an endothermic peak at 337.73 °C (enthalpy: 321.1 J/g) with an onset temperature of 314.88 °C, corresponding to the melting point of MCC. Mannitol displayed a sharp melting peak with onset at 166.03 °C and a transitional enthalpy value of 321.7 J/g. Owing to the extremely high melting point of colloidal silicon dioxide that occurs at 1600 °C, no phase transitions or peaks were observed in the range of 50–250 °C [25].

The sharp melting peak of carvedilol remained consistent when combined with magnesium stearate, suggesting compatibility between the two components (Figure 1). However, the peak corresponding to the dehydration of magnesium stearate has shifted to a peak temperature of 102.93 °C (enthalpy: 29.51 J/g) from 87.92 °C (enthalpy: 19.70 J/g). The third endothermic peak at 127.16 °C (enthalpy: 11.53 J/g), indicating the fusion of constituent fatty acids also shifted from a peak temperature of 115.03 °C (enthalpy: 274.7 J/g) with reduced enthalpy value. Such changes may be due to the different polymorphic forms of magnesium stearate in which melting points vary depending on the concentration of the specific polymorphic form present [28]. It may be possible for a certain polymorphic form to be present at a higher concentration in the mixture as compared to the individually screened magnesium stearate. Additionally, due to the similar melting points of both components in the mixture, it is possible that less energy was required to fulfil the fusion of constituent fatty acids in magnesium stearate since the melting of carvedilol occurred first and therefore less energy was needed to fulfil the later fusion. No substantial peak shifts, appearance of new peaks, or changes in enthalpy values were observed between carvedilol and mannitol, MCC and CSD mixtures, thereby demonstrating compatibility (Figure 1).

### 3.2. Pre-Compression Material Characterisation

For low concentration drugs, as in the case of carvedilol (1 and 4% *w*/*w*), the success of direct compression is dictated by the excipients to ensure content uniformity, flow, and compressibility [29]. It is essential for the powder to flow evenly, ensuring the right amount of powder enters the tabletting die, allowing consistent tablet weight and content uniformity.

The cohesiveness of a material must be determined before blending to evaluate flow properties. Cohesive materials have an average particle size of less than 50 μm and tend to aggregate, leading to intermitted flow [30]. On the other hand, non-cohesive materials have a larger particle size (>50 μm) and are expected to have good flow properties for successful tableting via the direct compression method [31]. The volume mean diameter (VMD) of carvedilol was 24.68 ± 1.97 μm, suggesting its cohesive nature and tendency to aggregate (Table 3). MCC on the other hand displayed a larger VMD of 84.20 ± 0.40 μm, suggesting non-cohesive behaviour. Larger particles, as in the case of MCC and mannitol (70.92 ± 3.27 μm) exhibit better flow compared to materials with smaller particle sizes due to the reduced surface area available for inter-particulate interactions [32]. Magnesium stearate showed a VMD of 17.4 ± 1.96 μm indicating its cohesive nature. However, magnesium stearate when mixed, forms a boundary layer around particles in the formulation, resulting in reduced friction between particles and optimising the flow of the powder blend [33]. The hydrophobic nature of magnesium stearate also reduces inter-particulate interactions by minimising wan der Waals forces, thereby improving flow [34].

The angle of repose (AOR) indicated carvedilol to possess poor flow (46.47°) (Table 4), which is expected because the small particle size of carvedilol means there is a greater surface area available for interaction between carvedilol particles. On its own, magnesium stearate showed passable flow (40.33°) however, it is well known that lubricants improve flow properties when added to powders. MCC and mannitol showed excellent and fair flow properties (23.73° and 32.97°). It is important for MCC and mannitol to possess good flow properties as they will dictate the overall flow of the formulation blend, since they are at high concentrations and make the bulk of the powder blend. After incorporating CSD, the angle of repose for formulations 1 and 2 both displayed excellent flowability. Flow aids enhance flow by adsorbing onto the particles within the formulation, thereby increasing surface roughness of host particles, resulting in reduced van der Waals forces [35].

According to Carr’s index and Hausner ratio, mannitol exhibited very poor flow (33.33% and 1.5) whereas MCC shows passable flow (23.08% and 1.3) (Table 4). These results are in disagreement with results presented by the angle of repose. This may be due to the shape of the particles, where angular particles, as in the case of MCC, may initially pack loosely but when subjected to force result in significant repacking, resulting in a higher Hausner ratio, leading to an untrue representation of actual flow property [36]. The fibrous and irregular particle shape of MCC may also contribute to its inter-particulate void volume, leading to a higher volume to mass ratio [20,37]. The same can be said for F1 and F2 where flow evaluation based on CI and HR indicates poor flow, where in fact, both formulation blends actually possess excellent flowability.

### 3.3. Optimising the Process of Blending for Carvedilol to Improve Content Uniformity

Mixing of particulate solids is an important process step in achieving homogenous blends and key to assuring that each resulting unit dose possesses the specified amount of drug to achieve effective therapeutic levels. For low concentration drugs, as in the case of carvedilol, the success of direct compression is dictated by the excipients which are incorporated into the powder blend, with an aim to optimise flow and compressibility. It is essential for the powder to flow evenly, ensuring that the right amount of homogeneous powder enters the tableting die, ensuring consistent tablet weight and content uniformity [38].

It was expected that longer mixing time would provide better content uniformity, since the API particles would have more contact time with other particles, increasing the chances of collision and resulting in improved homogeneity [39,40]. However, this was not the case as an increase in mixing time to 15 min increased the percentage recovery of carvedilol. The increase in mixing time may have led to the segregation of carvedilol particles, owing from its particle size, density, and cohesive nature [41]. Denser particles, as in the case of carvedilol, promote de-mixing and non-homogeneity in the mix, since dense particles consistently move downwards and settle at the base of the powder blend. Additionally, longer blending times impose greater particle-particle collisions, resulting in increased particle mobility and dilation of the powder bed. Blending optimisation requires careful consideration of the order of addition of excipients together with fine tuning process parameters. A total mixing time of 5 min at a speed of 250 rotations per minutes and with MCC added first provided optimised conditions for successful tableting and content uniformity (Table 5). MCC exhibits high surface roughness, enabling it to improve content uniformity of fine materials by promoting particle interlocking and acting as a host with multiple cavities for API entrapment [42]. Additionally, a high surface could potentially result in fewer adhesive interactions between particles, thereby improving flow and increasing the chances of achieving a homogenous blend [43].

### 3.4. ODMT Evaluation

Both formulations produced a balance between mechanical strength and fast disintegration times (Table 6). Friability was below the 1% European pharmacopeia limit for both formulations, suggesting minimal residual mass loss during transportation and handling. Formulations disintegrated within the recommended US Food and Drug Administration (FDA) disintegration time limit of 30 s (and well within the European Pharmacopoeia limit of 3 min) [44,45]. Fast disintegration times allows tablets to be safely administered to children either directly into the mouth or suspended on a spoon prior to administration. The British Pharmacopeia (BP) states that in order for the dosage form to fulfil the requirement, the content of carvedilol in each unit dose must be between 95–105% the stated amount [46]. The percentage recovery of carvedilol tablets (F1 and F2) was within the specified values, suggesting appropriate therapeutic concentrations and safety.

### 3.5. ODMT Evaluation–Biorelevant Dissolution Studies

Carvedilol is a weakly basic compound with a pKa value of 7.8, exhibiting the distinctive pH-dependent solubility profile of a weak base, with low solubility in dissolution media of high pH values and high solubility in media of low pH values due to changes in ionisation [11] and as such, the solubility of carvedilol is dependent upon the specific biorelevant media characteristics. The range of physiological pH and compositions of different parts of the gastrointestinal (GI) tract may influence solubility and consequently affect the dissolution rate and total extent of carvedilol release.

All adult and paediatric biorelevant media prepared for dissolution testing are categorised as level II media (biorelevant) that mimic various parts of the GI tract in regards to adjusted osmolality, pH, buffer capacity, bile components, and phospholipid concentration, and therefore, are superior to conventional USP/BP dissolution methods as they better reflect the solubilisation capacity of adult and paediatric GI fluids both during the fed and fasted state [47].

**Figure 2:**
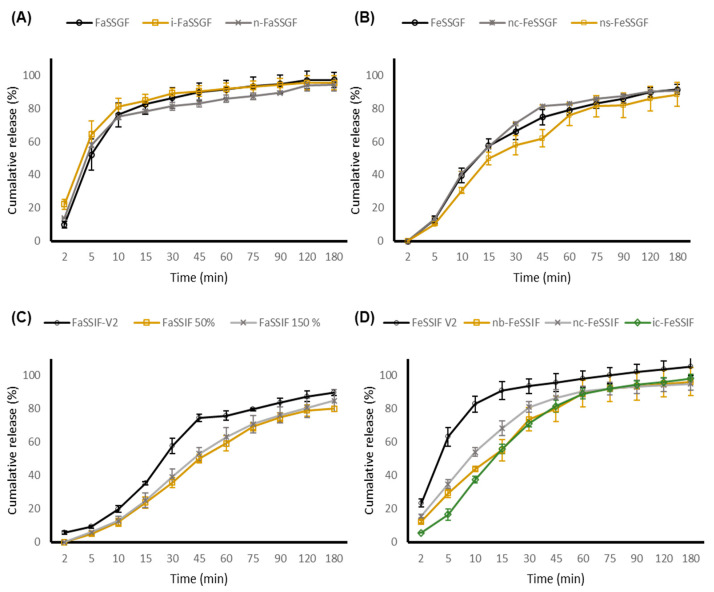
Dissolution profile of carvedilol 0.5 mg ODMT in adult and paediatric biorelevant media. Data presented as mean ± standard deviation (*n* = 6). (**A**) FaSSGF = Adult Fasted-state Simulated Gastric Fluid, (**B**) FeSSGF = Adult Fed-state Simulated Gastric Fluid, (**C**) FaSSIF-V2 = Adult Fasted-state Simulated Intestinal Fluid, and (**D**) FeSSIF = Fed-state Simulated Intestinal Fluid. i = infant, n = neonate, c = cow-based milk formula, s = soy-based milk formula, and b = breast fed.

**Figure 3:**
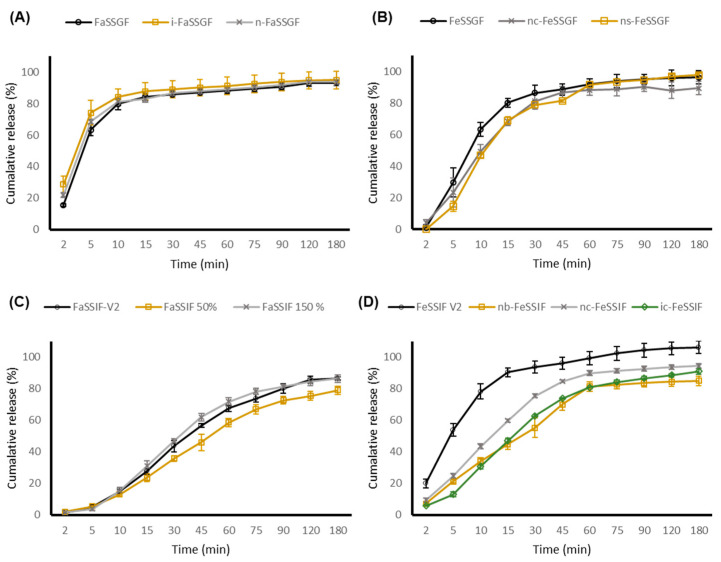
Dissolution profile of carvedilol 2 mg ODMT in adult and paediatric biorelevant media. Data presented as mean ± standard deviation (*n* = 6). (**A**) FaSSGF = Adult Fasted-state Simulated Gastric Fluid, (**B**) FeSSGF = Adult Fed-state Simulated Gastric Fluid, (**C**) FaSSIF-V2 = Adult Fasted-state Simulated Intestinal Fluid, and (**D**) FeSSIF = Fed-state Simulated Intestinal Fluid. i = infant, n = neonate, c = cow-based milk formula, s = soy-based milk formula, and b = breast fed.

The initial rate of release of carvedilol from the novel ODMTs in a pre-prandial state was immediate, with the carvedilol percentage release exceeding 80% after the first 10 min (Figure 2 and Figure 3). The absorption of carvedilol is rapid, where maximum serum concentration is achieved between 1 to 2 h post administration [48]. Both formulations approach plateau levels approximately after 45 min within all fasted state simulated gastric media, with no significant differences observed between the adult and age-specific biorelevant media (*p* value > 0.05). It was expected that the dissolution rate to decrease in adult and paediatric FeSSGF due to the higher pH values of 5 and 5.7. This was true as the percentage release of carvedilol was only about 30–40% after the first 10 min. Nonetheless, no significant difference between adult and paediatric biorelevant media was observed.

Although the fasted state of the gastric media is ideal for rapid and complete dissolution, it has been advised that heart failure patients take their medication with food to avoid a sharp increase in plasma concentration and limit the risk of orthostatic hypotension [49]. Due to regular feeding intervals, neonates and young infants are generally always in a continuous state of post prandial, and therefore are most likely would follow the dissolution trend as depicted in FeSSGF, where drug release is gradual, thereby restricting a sudden increase in drug plasma concentrations and limiting the risk of observing adverse effects.

On the other hand, a clinically relevant dissolution profile between FeSSIF-V2 and paediatric FeSSIF was observed, where both ODMT formulations displayed a faster dissolution rate and an overall greater extent of percentage carvedilol release in FeSSIF-V2. This may be owing to the larger concentration of bile salt (sodium taurocholate) and lipid (lecithin) in FeSSIF-V2. Lecithin enhances the solubility of drugs through complexation, whilst sodium taurocholate holds the ability to produce micelles. Other than micellar solubilisation, bile salts may alter drug polarity and therefore solubility through ion pairing, when interacting with drug molecules [50]. Based upon our findings, there was no real difference between ODMT dissolution profiles in adult and paediatric biorelevant media (apart from FeSSIF). Furthermore, paediatric GI physiology and factors affecting paediatric permeability (microbiota, transport systems, and metabolising enzymes) are still not fully understood [51,52].

**Figure 4:**
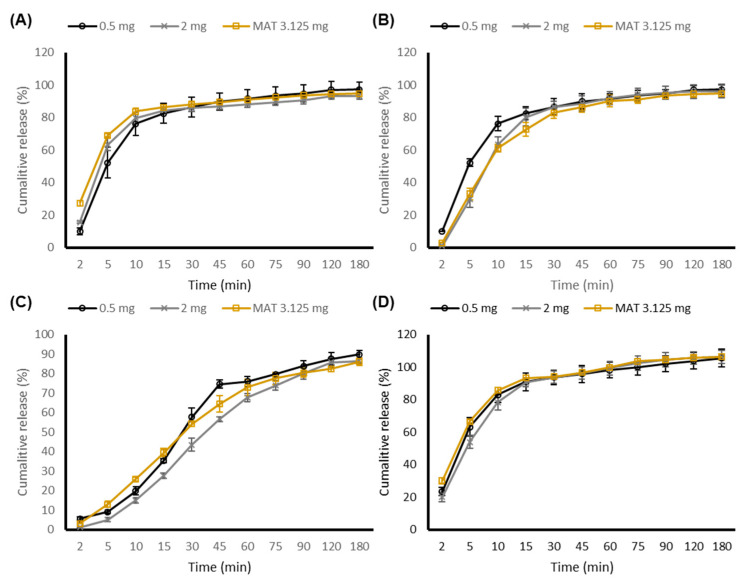
Dissolution profile comparison between carvedilol 0.5 mg ODMT, 2 mg ODMT, and marketed 3.125 mg tablet in adult and paediatric biorelevant media. Data presented as mean ± standard deviation (*n* = 6). MAT = Marketed adult tablet. (**A**) FaSSGF, (**B**) FeSSGF, (**C**) FaSSIF-V2, and (**D**) FeSSIF-V2.

Figure 4 compares the in vitro drug performance of both the 0.5 mg and 2 mg ODMT formulation against a marketed carvedilol tablet (MAT). Since the dose of carvedilol in paediatrics is variable, 3.125 mg (lowest strength available) marketed carvedilol tablet was selected as the control. Although not labelled as an ODT, the marketed tablet contains povidone and crospovidone type A and B, all of which display disintegrating and dissolution and solubility-enhancing properties. The inclusion of such excipients would assume a faster dissolution rate however, this was not the case, as all three formulations depicted similar dissolution profiles across the GI tract with no significant (*p* > 0.05) differences observed. The choice of excipients and concentrations utilised, alongside fine-tuned production parameters for the manufactured carvedilol ODMT, ensured a robust dissolution profile, comparable to the marketed formulation without including dedicated functional disintegrants. Tablet disintegration is a prerequisite for dissolution, where orodispersible formulations must disintegrate rapidly however, the use of superdisintegrants in and as a model formulation constituent is not preferred, since many are incompatible with various drugs compounds, moisture sensitive (leading to instability), and lack toxicity data in both adults and paediatrics [25]. Upon comparison with the marketed tablet, reformulation to an ODMT is likely to not have any detrimental effect clinically, and at the same time demonstrated superiority due the advantage of being a much more acceptable dosage form with an ability for flexible dosing.

## 4. Conclusions

Two strengths of carvedilol ODMT formulations were developed using an excipient composition and load that is appropriate for paediatric use. The novel ODMTs displayed all relevant features of an age-appropriate formulation, including enhanced dose flexibility, palatability, excipient safety, and concordance to development and ability for safe administration. Dissolution profiles observed were robust and comparable to the marketed formulation across various part of the GI tract in both the fed and fasted state, signifying appropriate efficacy, quality, and performance. No significant difference in dissolution profiles between adult and paediatric biorelevant media were established. The systematic selection of drug candidates listed within the EMA inventory of paediatric therapeutic needs, alongside paediatric specific formulation approach, provides an encouraging starting point that ultimately aims to increase the availability of more age-appropriate formulations for children through PUMA applications.

## Figures and Tables

**Table 1:** Formulation composition for 500 and 2000 µg carvedilol mini ODT production.

Ingredient	F1 (0.5 mg Tablet) Concentration% *w*/*w*	F2 (2 mg Tablet) Concentration% *w*/*w*
Mannitol	83.25	80.25
MCC	15	15
Carvedilol	1	4
Colloidal silica dioxide	0.25	0.25
Magnesium stearate	0.5	0.5

**Table 2:** Composition of adult and paediatric biorelevant media used in solubility and dissolution experiments [21,22]. FaSSGF = Fasted-state Simulated Gastric Fluid, FeSSGF = Fed-state Simulated Gastric Fluid, FaSSIF = Fasted-state Simulated Intestinal Fluid, FeSSIF = Fed-state Simulated Intestinal Fluid, i = infant, n = neonate, c = cow-based milk formula, s = soy-based milk formula, and b = breast fed.

Pre-Prandial Biorelevant Gastric Media Composition				
Composition	Adult (FaSSGF)	Infant (i-FaSSGF)		Neonate (n-FaSSGF)
Sodium chloride (mM)	34.2	34.2		34.2
Sodium taurocholate (μM)	80	60		20
Lecithin (μM)	20	15		5
Pepsin (mg/mL)	0.1	0.025		0.015
HCl/NaOH qs	pH 1.6	pH 1.6		pH 1.6
pH	1.6	1.6		1.6
Osmolarity (mOsm/kg)	120.7 ± 2.5	120.7 ± 2.5		120.7 ± 2.5
**Post-prandial biorelevant gastric media composition**				
**Composition**	**Adult (FeSSGF)**	**Neonate-Cow (nc-FeSSGF)**		**Neonate-Soy (ns-FeSSGF)**
Sodium chloride (mM)	34.2	34.2		34.2
Acetic acid (mM)	17.12	7.25		7.25
Sodium acetate (mM)	29.75	64.65		64.65
Milk:buffer	1:1	1:1		1:1
HCl/NaOH qs	pH 5	pH 5.7		pH 5.7
pH	5	5.7		5.7
Osmolarity (mOsm/kg)	400	340		240
Buffering capacity (mmol/L/pH)	25	15		15
**Pre-prandial biorelevant intestinal media composition**				
**Composition**	**Adult (FaSSIF-V2)**	**FaSSIF-50%**		**FaSSIF-150%**
Sodium hydroxide (mM)	34.8	34.8		34.8
Sodium taurocholate (mM)	3	1.5		4.5
Lecithin (mM)	0.2	0.1		0.3
Sodium chloride (mM)	68.62	68.62		68.62
Maleic acid (mM)	19.12	19.12		19.12
HCl/NaOH qs	pH 6.5	pH 6.5		pH 6.5
Osmolarity (mOsm/kg)	180 ± 10	180 ± 10		180 ± 10
Buffering capacity (mmol/L/ph)	10	10		10
**Post-prandial biorelevant intestinal media composition**				
**Composition**	**Adult (FeSSIF** **-** **V2)**	**Neonate-breast fed (nb** **-** **FeSSIF)**	**Neonate-cow formula (nc** **-** **FeSSIF)**	**Infant-cow formula (i** **-** **FeSSIF)**
Sodium hydroxide (mM)	81.65	81.65	81.65	81.65
Sodium taurocholate (mM)	10	2.5	2.5	7.5
Lecithin (mM)	2	0.5	0.5	1.5
Sodium chloride (mM)	125.5	95	111.73	107.35
Maleic acid (mM)	55.02	55.02	55.02	55.02
Glyceryl monooleate (mM)	5	5	6.65	5
Sodium monooleate (mM)	0.8	0.8	1.06	0.8
HCl/NaOH qs	pH 5.8	pH 5.8	pH 5.8	pH 5.8
Osmolarity (mOsm/kg)	300 ± 10	330 ± 10	330 ± 10	390 ± 10
Buffering capacity (mmol/L/ph)	25	25	25	25

**Table 3:** Particle size analysis of starting materials and optimised formulation blends (formulations 1 and 2).

Sample	VMD (μm)
Mannitol	70.92 ± 3.27
MCC	84.20 ± 0.40
Carvedilol	24.68 ± 1.97
Magnesium stearate	17.49 ± 1.96
F1	77.50 ± 0.60
F2	77.18 ± 1.17

**Table 4:** Angle of repose, Carr’s index (CI), Hausner ratio (HR), and evaluation based on CI and HR.

Sample	AOR (°)	CI (%)	HR	Evaluation Based on CI and HR
Mannitol	32.97	33.33	1.50	Very, very poor
MCC	23.73	23.08	1.30	Passable
Carvedilol	46.47	57.89	2.38	Very, very poor
Magnesium stearate	40.33	44.41	1.80	Very, very poor
F1	15.97	27.78	1.38	Poor
F2	16.36	27.78	1.38	Poor

**Table 5:** Effect of processing parameters and blending order on percentage recovery of carvedilol. (F1) 250 rpm for 5 min, (F2) 250 rpm for 10 min, (F3) 100 rpm for 5 min, (F4) 100 rpm for 10 min, (F5) 100 rpm for 15 min, (F6) 250 rpm for 15 min, (F7) 250 rpm for 5 min (MCC added first), and (F8) 100 rpm for 10 min (MCC added first). Data presented as mean ± standard deviation (*n* = 10).

Blend	% Carvedilol Recovery	RSD
1	111.8 ± 7.4	6.61
2	102.4 ± 7.4	7.27
3	104.9 ± 8.2	7.85
4	93.7 ± 5.9	6.31
5	148.6 ± 12.3	8.31
6	140.6 ± 9.2	6.56
7	103.5 ± 3.2	3.06
8	104.4 ± 5.3	5.05

**Table 6:** Tablet properties. Hardness value and disintegration time presented as mean ± standard deviation (*n* = 3). Content uniformity presented as mean ± standard deviation (*n* = 10).

Formulation	Hardness (N)	Friability (% Loss)	Disintegration (s)	Content Uniformity (%)
F1 (0.5 mg ODMT)	35.20 ± 11.72 N	0.22	11.2 ± 3.2	102.2 ± 2.9
F1 (2 mg ODMT)	33.62 ± 7.39 N	0.23	10.6 ± 2.4	101.7 ± 3.6

## Data Availability

Not Applicable.
